# Cross-sectional associations between prevalent vertebral fracture and pulmonary function in the sixth Tromsø study

**DOI:** 10.1186/1471-2318-13-116

**Published:** 2013-10-29

**Authors:** Bente Morseth, Hasse Melbye, Svanhild Waterloo, Marte R Thomassen, Marijke J Risberg, Nina Emaus

**Affiliations:** 1 Department of Community Medicine, University of Tromsø, Tromsø, Norway; 2 Regional Centre for Sport, Exercise and Health, Faculty of Health Sciences, University of Tromsø, Tromsø, Norway; 3 General Practice Research Unit, Department of Community Medicine, University of Tromsø, Tromsø, Norway; 4 Department of Occupational and Environmental Medicine, University Hospital of North Norway, Tromsø, Norway; 5 University Hospital of North Norway, Tromsø, Norway; 6 Department of Health and Care Sciences, University of Tromsø, Tromsø, Norway

**Keywords:** Vertebral fractures, Pulmonary function, Lung function, Population study, Epidemiology, Elderly

## Abstract

**Background:**

Persons with vertebral fracture may have reduced pulmonary function, but this association has not been much studied. The aim of this cross-sectional study was therefore to examine the relationship between vertebral fracture and pulmonary function in a general, elderly population.

**Methods:**

Vertebral morphometry was used for vertebral fracture assessment in 2132 elderly men (n = 892) and women (n = 1240) aged 55 to 87 years in the population-based Tromsø Study 2007–08. Pulmonary function was examined by spirometry. Pulmonary function was expressed as FVC% predicted, FEV_1_% predicted, and FEV_1_/FVC% predicted values, adjusted FVC, FEV_1_, and FEV_1_/FVC, and obstructive and restrictive ventilatory impairment. Vertebral fracture was classified according to appearance, number, severity, and location of fractures. Associations were analyzed using general linear and logistic models.

**Results:**

FVC% predicted and FEV_1_% predicted values were not associated with vertebral fracture (P > 0.05), whereas FEV_1_/FVC% predicted ratio was associated with both prevalent fracture, number of fractures, severity of fractures, and fracture site in men (P < 0.05), but not in women. When FVC, FEV_1_, and FEV_1_/FVC values were adjusted for multiple covariates, we found no significant association with vertebral fracture. Obstructive and restrictive ventilatory impairment was not associated with prevalent vertebral fracture.

**Conclusions:**

In conclusion, this study did not confirm any clinically relevant associations between prevalent vertebral fracture and ventilatory impairment in elderly individuals.

## Background

Osteoporotic fractures represent a major health concern among elderly people, and the highest incidences are found in Scandinavia [1-5]. Much attention has been directed toward the negative outcomes of hip fractures [6]; however, vertebral fractures are also associated with increased morbidity [7] and mortality [8-10]. Progressive collapse of the fractured vertebral body often results in kyphosis, height loss, chronic pain, and possibly impaired respiratory function [11,12]. Furthermore, vertebral fractures are related to a high annual number of additional vertebral fractures [13]. Previous patient-control data provide some evidence for an association between vertebral fracture or other measures of kyphosis and pulmonary function [11,14-17]. However, a recent systematic review including four case–control studies concluded that although patients with vertebral fracture demonstrate a modest reduction in pulmonary measures, evidence for an association is limited [11]. The limited number of studies with small sample sizes and predominantly female subjects call for further research in this area. We therefore examined whether presence of vertebral fracture was associated with measures of pulmonary function in a larger, general population of elderly men and women.

## Methods

### Design and subjects

The present study utilizes data from the 2007–08 Tromsø Study (Tromsø 6). The Tromsø Study is a longitudinal, population-based, multi-purposed health study with six repeated surveys between 1974 and 2008, conducted in the municipality of Tromsø, Northern Norway [18]. The Tromsø 5 and 6 surveys were conducted in two phases, with the most basic examination in phase 1 (height, weight, blood pressure, blood samples, and questionnaires), followed by more extensive examinations for random sub-samples of the cohort in phase 2 [19]. The Tromsø Study was approved by the Norwegian Data Inspectorate and the Regional Committee of Research Ethics. All participants signed a written informed consent.

A total of 19 762 subjects were invited to participate in Tromsø 6, and 12 984 (65.7%) of the invited subjects attended Tromsø 6, phase 1. Among the participants in phase 1, 11 484 subjects were invited to participate in phase 2. Of the 7307 subjects attending Tromsø 6, phase 2, only those with bone mineral density (BMD) measurement from the previous Tromsø 5 (2001–02) were invited for a dual X-ray (DXA) BMD measurement of the hip, i.e. a dual femur scan (n = 3854). Among these, lateral vertebral fracture assessment (VFA) was performed in a randomly selected group (n = 2894). Seven blurred VFA scans were excluded, leaving 2887 persons with valid VFA measurements.

**Figure 1:**
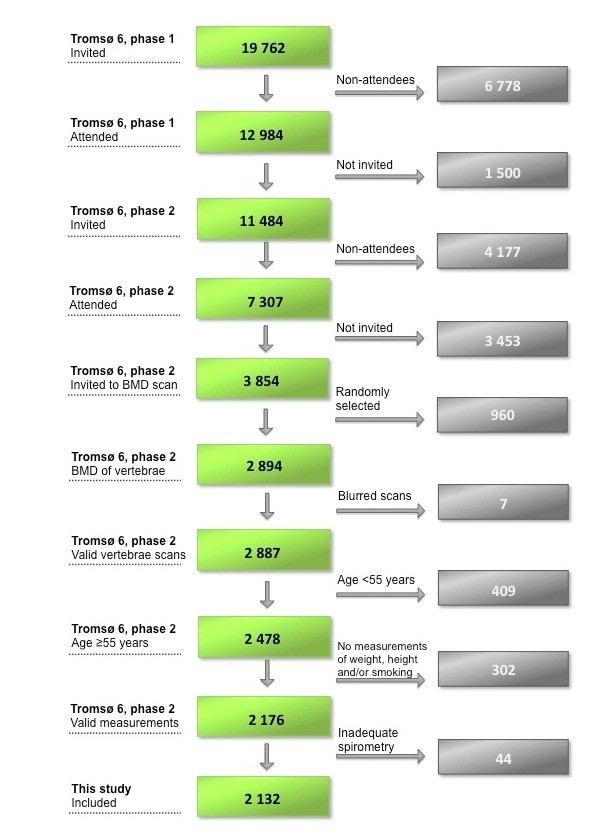
Flowchart of the study.

In the present study, we selected subjects aged ≥ 55 years with valid measurements of vertebral fractures (n = 2478), weight and height (n = 2221), and/or smoking habits (n = 2178), which left 2176 subjects for analyses (Figure 1). After exclusion of 13 (1.4%) men and 31 (2.4%) women with inadequate spirometry, the study comprised 2132 subjects. For the multiple adjusted models, we further excluded subjects with missing values for hip BMD (n = 171), physical inactivity (n = 277), and/or hormone drugs (n = 128 women), leaving 1674 persons (770 men and 904 women) for inclusion in the adjusted model.

### Assessment of pulmonary function

At the spirometry station in Tromsø 6, phase 2, recent symptoms of common cold, bronchitis, or other airway infections during the last 7 days were obtained through a short questionnaire [20]. Immediately following, spirometry was performed by trained technical staff with the use of a “Sensor Medics Vmax Encore 20” spirometer, following ATS/ERS criteria [21] with the subject seated. The quality of the spirometry has been described previously [22], reporting that both inter- and intra-observer agreements were close to optimal. Forced vital capacity (FVC) and forced expiratory volume in one second (FEV_1_) were measured with the subject performing a maximum inspiration followed by a complete forceful expiration.

### Ascertainment of vertebral fracture

**Figure 2:**
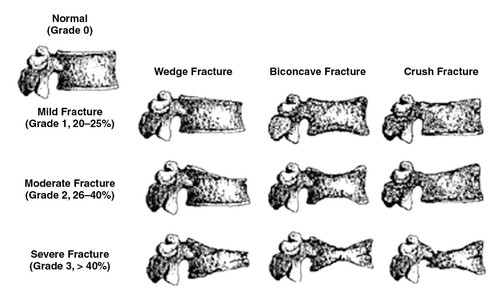
**Diagram of a semiquantitative method for diagnosing osteoporotic vertebral fractures.** The semiquantitative method was developed for identification of osteoporotic vertebral fractures based on measurement of vertebral height, identifying the anterior, middle, and posterior heights of each vertebra. Depending on their relative relations according to a given reference, three types of fractures are identified: wedge, biconcave, and compression, according to three degrees of severity, ranging from mild through moderate to severe. The wedge fractures are characterized by deformed structure of the anterior part of the vertebrae, the biconcave of the middle part, and the compression of the total vertebrae. Reprinted from Lenchik et al. [25] with permission from the American Journal of Roentgenology.

Vertebral fracture was ascertained by VFA according to a standard set by GE Lunar Prodigy, Lunar Corp., Madison, USA; software version 12.20 [23]. VFA is a semi-quantitative method developed for identification of osteoporotic vertebral fractures based on measurement of vertebral height, identifying the anterior, middle, and posterior heights of each vertebra. Depending on their relative relations according to a given reference, the software identifies three types of fractures: wedge, biconcave, and compression, according to three degrees of severity, ranging from mild through moderate to severe [24,25] (Figure 2). The wedge fractures are characterized by deformed structure of the anterior part of the vertebrae, the biconcave of the middle part, and the compression of the total vertebrae. In our dataset, only 1% of the deformities were identified as being mild, thus persons with mild fracture were moved to the “moderate” category.

Specially trained technicians did the scanning according to the standardized protocol, and quality assessment of the identified vertebrae was performed manually afterwards. For precision analysis of the VFA, data from a random sample of 50 participants was reanalyzed. The mean intra-class correlation coefficient was 0.82, 0.79, 0.82, and 0.84 for anterior, middle, posterior, and average height, respectively, all vertebrae considered [26]. At the vertebrae with highest frequency of present deformity, exemplified by 7^th^ and 12^th^ thoracic vertebrae, the mean intra-class correlation coefficient was 0.86 (range 0.77 to 0.92).

### Assessment of covariates

Prior to the examinations, all participants responded to a self-administered questionnaire concerning health related topics including questions about past and current smoking habits, cardiovascular disease (angina pectoris, myocardial infarction, or cerebral stroke), and physical activity. Women also reported use of systemic estrogens (hormone therapy for menopausal symptoms). Use of inhaled and systemic corticosteroids was defined from self-report of brand names belonging to ATC groups R03BA and R03AK, and H02AB, respectively. At the physical examination, height and weight were measured to the nearest centimeter and half-kilogram, respectively, with subjects wearing light clothing and no shoes. BMD of the total hip was measured in g/cm^2^ by the same GE Lunar Prodigy DXA scanner used for the vertebral morphometry.

### Statistical analyses

Possible associations between presence of vertebral fracture as exposure and pulmonary function measures as outcome were examined using general linear models (linear regression analyses and analysis of variance; ANOVA and ANCOVA). Pulmonary function was expressed as 1) predicted percentages of FVC, FEV_1_, and FEV_1_/FVC values, 2) multiple adjusted FVC, FEV_1_, and FEV_1_/FVC values, and 3) obstructive and restrictive ventilatory impairment.

Each vertebral fracture was classified according to presence, number, severity, and location of fractures. Presence of vertebral fracture was confirmed if at least one fracture was present at any level of the vertebrae. Airways restriction was defined as FVC and FEV_1_ less than 80% of predicted values and FEV_1_/FVC ≥0.7. As main criterion, obstruction was defined as FEV_1_/FVC <0.7, with mild obstruction defined as FEV_1_% predicted ≥80%, moderate obstruction as FEV_1_% predicted <80% and ≥50%, and severe obstruction defined as FEV_1_% predicted <50%.

Calculations of predicted values were based on the equations published by Langhammer et al. [27] for a Norwegian adult population, which are based on age and current body height. Since reduced height due to vertebral fractures leads to decreased predicted values of spirometry, and accordingly increased % predicted values, we repeated the analyses using previous height in 1994–95 instead of current height in a subsample (n = 2058).

In the multiple adjusted model, FVC, FEV_1_ and FEV_1_/FVC ratio were controlled for age, smoking habits, weight, current height, physical activity, use of corticosteroids and hormones for menopause, total hip BMD, self-reported cardiovascular diseases (myocardial infarction, cerebral stroke, or angina pectoris) and chronic lung diseases (asthma or chronic bronchitis/emphysema/chronic obstructive pulmonary disease (COPD)). Test for multicollinearity between independent variables did not reveal high correlation between any of the covariates. As one could expect, both women and men showed highest correlation between height and weight (0.3-0.5, P < 0.001) and between lung disease (CLD) and use of corticosteroids (0.5-0.6, P < 0.001). Tolerance was high (>0.6).

Finally, we calculated age-adjusted and multiple adjusted odds for experiencing airways restriction and obstruction in relation to prevalence of vertebral fracture.

Two-sided *P* values <0.05 were considered statistically significant. All analyses were performed using IBM SPSS Statistics, version 19.

## Results

The study comprised 892 men and 1240 women between 55 and 87 years, with a mean age of 67.8 (SD 7.5) years. Vertebral fracture was present in 121 (13.6%) men and 161 (13.0%) women. As shown in Table 1, subjects with vertebral fracture were older and had lower total hip BMD than those without (P < 0.001). In addition, women with vertebral fracture were shorter and weighed less than women without fracture (P < 0.05). Corticosteroid use and pulmonary disease (men) and frequency of cardiovascular disease (women) were higher in subjects with fracture (P < 0.001). Men and women with vertebral fracture had lower absolute FVC (P ≤ 0.014), FEV_1_ (P ≤ 0.001), and FEV_1_/FVC ratio (P ≤ 0.046) than those with no fracture.

**Table 1:** Characteristics of the study population by gender and vertebral fracture

**Characteristics**	**Men (n = 892)**			**Women (n = 1240)**		
	**No vertebral fracture (n = 771)**	**Vertebral fracture (n = 121)**	***P***	**No vertebral fracture (n = 1079)**	**Vertebral fracture (n = 161)**	***P***
Age (years)	67.4 (7.6)	70.4 (7.5)	<0.001	67.3 (7.1)	71.3 (7.6)	<0.001
Body height (cm)	175.1 (6.5)	174.2 (6.9)	0.182	162.1 (6.1)	160.3 (7.1)	0.001
Body weight (kg)	83.9 (12.1)	82.9 (11.7)	0.371	71.0 (12.4)	68.8 (12.4)	0.039
Hip bone mineral density (BMD) (g/cm^2^)^a^	1.027 (0.142)	0.973 (0.161)	<0.001	0.901 (0.130)	0.830 (0.115)	<0.001
Smoking			0.491			0.421
Current	13.6% (105)	17.4% (21)		17.5% (189)	19.3% (31)	
Past	61.6% (753)	57.0% (69)		41.5% (448)	36.0% (58)	
Never	24.8% (191)	25.6% (31)		41.0% (443)	44.7% (72)	
Physical inactivity^b^	20.4% (141)	17.4% (19)	0.661	19.7% (181)	18.8% (26)	0.947
Cardiovascular disease^c^	22.6% (174)	24.0% (29)	0.733	10.1% (109)	15.5% (25)	0.038
Lung disease^d^	11.2% (86)	18.2% (22)	0.028	14.8% (160)	12.4% (20)	0.421
Hormone drugs for menopause^e^				7.7% (76)	4.6% (6)	0.193
Corticosteroid use	5.8% (45)	11.6% (14)	0.018	8.3% (90)	9.3% (15)	0.678
FVC (liter (SE))	4.20 (0.81)	4.00 (0.86)	0.014	2.95 (0.56)	2.77 (0.64)	<0.001
FEV_1_ (liter (SE))	3.05 (0.70)	2.82 (0.79)	0.001	2.18 (0.48)	2.02 (0.55)	<0.001
FEV_1_/FVC (% (SD))	72.5 (8.7)	69.9 (10.5)	0.003	73.8 (7.4)	72.5 (8.4)	0.046

The Tromsø Study 2007-08.

Values are mean (SD) or % (n).

^a^n = 860 men and 1152 women.

^b^n = 799 men and 1057 women.

^c^Cerebral stroke, myocardial infarction, or angina pectoris.

^d^Asthma, chronic bronchitis, emphysema, or COPD.

^e^n = 1112 women.

### Predicted FVC%, FEV_1_%, and FEV_1_/FVC% values

FVC% predicted and FEV_1_% predicted did not differ significantly between subjects with and without fracture. Men with vertebral fracture had lower FEV_1_/FVC% predicted than men with no vertebral fracture (P = 0.003) (Table 2).

**Table 2:** Associations between vertebral fracture and lung function

	**Pulmonary function (mean (SD) or (SE))**					
	**Men**			**Women**		
	**No Vertebral fracture (n = 771)**	**Vertebral fracture (n = 121)**	***P***	**No vertebral fracture (n = 1079)**	**Vertebral fracture (n = 161)**	***P***
FVC% predicted^a^	98.2 (17.4)	97.2 (17.6)	0.522	101.2 (15.8)	102.2 (18.2)	0.475
FEV_1_% predicted^a^	88.9 (16.9)	85.6 (21.3)	0.055	93.0 (17.4)	93.3 (20.9)	0.842
FEV_1_/FVC% predicted^a^	90.6 (10.8)	87.4 (13.0)	0.003	92.0 (9.1)	90.6 (10.4)	0.083
Adjusted^b^ values	(n = 665)	(n = 105)		(n = 800)	(n = 104)	
FVC (liter (SE))	4.22 (0.024)	4.23 (0.062)	0.951	2.98 (0.015)	3.01 (0.043)	0.560
FEV_1_ (liter (SE))	3.08 (0.021)	3.05 (0.054)	0.650	2.21 (0.013)	2.24 (0.037)	0.408
FEV_1_/FVC (SE)	0.73 (0.003)	0.72 (0.007)	0.291	0.74 (0.002)	0.74 (0.007)	0.557

The Tromsø Study 2007-08.

^a^Equation from Langhammer et al. [27].

^b^Adjusted for age, smoking habits, height, weight, physical inactivity, cardiovascular disease, lung disease, corticosteroids, hip BMD, hormones for menopause (women).

In men, FVC% predicted, FEV_1_% predicted, and FEV_1_/FVC% predicted ratio values decreased as number of vertebral fractures increased (P < 0.05) (Table 3). In women, no such trend was found for FVC% predicted and FEV_1_% predicted, whereas FEV_1_/FVC% predicted ratio was inversely related to number of vertebral fractures (P = 0.021). Women and men with 4 or more fractures had significantly lower FEV1% predicted than those without fracture (P ≤ 0.05).

**Table 3:** Associations between number of vertebral fracture and lung function

	**Pulmonary function (mean (SD) or (SE))**											
	**Men**						**Women**					
	**Number of vertebral fractures**						**Number of vertebral fractures**					
	**0 (n=771)**	**1 (n=83)**	**2 (n=26)**	**3 (n=9)**	**4-6 (n=3)**	***P trend***	**0 (n=1079)**	**1 (n=103)**	**2 (n=37)**	**3 (n=11)**	**4-6 (n=10)**	***P trend***
FVC% predicted^a^	98.2 (15.3)	97.9 (17.9)	99.5 (17.1)	90.0 (15.2)	80.1 (10.3)	0.047	101.2 (15.8)	102.6 (18.3)	101.7 (17.4)	107.6 (16.1)	94.1 (21.1)	0.137
FEV_1_% predicted^a^	88.9 (16.9)	87.2 (20.2)	84.3 (24.9)	79.6 (22.7)	70.8 (12.3)	0.007	93.0 (17.4)	94.2 (20.5)	93.1 (20.5)	94.2 (24.0)	83.9 (23.3)	0.437
FEV_1_/FVC% predicted^a^	90.6 (10.8)	88.8 (10.9)	83.2 (17.7)	87.2 (16.4)	87.8 (4.3)	0.002	92.0 (9.1)	91.2 (10.4)	91.1 (9.3)	86.0 (13.7)	88.0 (10.5)	0.021
Adjusted^b^ values	(n=665)	(n=74)	(n=22)	(n=7)	(n=2)		(n=800)	(n=65)	(n=26)	(n=5)	(n=8)	
FVC (liter (SE))	4.22 (0.024)	4.22 (0.073)	4.29 (0.133)	4.18 (0.237)	4.10 (0.442)	0.982	2.99 (0.015)	3.09 (0.053)	2.90 (0.084)	2.89 (0.192)	2.75 (0.154)	0.321
FEV_1_ (liter (SE))	3.08 (0.021)	3.07 (0.063)	2.94 (0.116)	3.24 (0.206)	3.12 (0.384)	0.747	2.21 (0.013)	2.32 (0.046)	2.15 (0.073)	2.04 (0.166)	2.08 (0.134)	0.476
FEV_1_/FVC (SE)	0.73 (0.003)	0.73 (0.009)	0.67 (0.016)	0.79 (0.028)	0.76 (0.053)	0.502	0.74 (0.002)	0.75 (0.008)	0.74 (0.013)	0.69 (0.029)	0.76 (0.023)	0.874

The Tromsø Study 2007-08.

^a^Equation from Langhammer et al. [27].

^b^Adjusted for age, smoking habits, height, weight, physical inactivity, cardiovascular disease, lung disease, corticosteroids, hip BMD, hormones for menopause (women).

Neither FVC% predicted nor FEV_1_% predicted were associated with fracture site (T4-T12 or L1-L4) (Table 4). FEV_1_/FVC% predicted was significantly lower in men and women with lumbar (L1-L4) fracture and in men with thoracic (T4-T12) fracture, compared with men and women with no fracture.

**Table 4:** Associations between vertebral fracture and lung function according to fracture site

	**Pulmonary function (mean (SD) or (SE))**									
	**Men**					**Women**				
	**No vertebral fracture (n = 771)**	**Fracture T4-T12 (n = 82)**	**Fracture L1-L4 (n = 39)**	***P *** **T4-T12**^**a**^	***P *** **L1-L4**^**a**^	**No vertebral fracture (n = 1079)**	**Fracture T4-T12 (n = 94)**	**Fracture L1-L4 (n = 67)**	***P *** **T4-T12**^**a**^	***P *** **L1-L4**^**a**^
FVC% predicted^b^	98.2 (15.3)	97.0 (17.5)	97.7 (18.1)	0.500	0.856	101.2 (15.8)	104.4 (17.4)	99.0 (18.7)	0.058	0.264
FEV_1_% predicted^b^	88.9 (16.9)	85.7 (21.4)	85.3 (21.5)	0.120	0.219	93.0 (17.4)	95.8 (19.5)	89.8 (22.3)	0.145	0.154
FEV_1_/FVC% predicted^a^	90.6 (10.8)	87.8 (13.6)	86.8 (11.8)	0.027	0.035	92.0 (9.1)	91.3 (9.6)	89.6 (11.4)	0.505	0.046
Adjusted^c^ values	(n = 665)	(n = 74)	(n = 31)			(n = 800)	(n = 60)	(n = 44)		
FVC (liter (SE))	4.22 (0.024)	4.18 (0.073)	4.34 (0.113)	0.569	0.296	2.99 (0.015)	3.10 (0.055)	2.89 (0.066)	0.045	0.141
FEV_1_ (liter (SE))	3.08 (0.021)	3.01 (0.063)	3.16 (0.098)	0.292	0.415	2.21 (0.013)	2.32 (0.048)	2.14 (0.057)	0.032	0.212
FEV_1_/FVC (SE)	0.73 (0.003)	0.72 (0.009)	0.73 (0.014)	0.240	0.857	0.74 (0.002)	0.75 (0.008)	0.74 (0.010)	0.405	0.960

The Tromsø Study 2007-08.

^a^Versus no fracture.

^b^Equation from Langhammer et al. [27].

^c^Adjusted for age, smoking habits, height, weight, physical inactivity, cardiovascular disease, lung disease, corticosteroids, hip BMD, hormones for menopause (women).

Severity of fracture did not influence FVC% predicted, FEV_1_% predicted, or FEV_1_/FVC % predicted, except in men with severe fracture, who had lower FEV_1_/FVC % predicted than men with no fracture (P = 0.004) (Table 5).

**Table 5:** Associations between vertebral fracture and lung function according to severity of fracture

	**Pulmonary function (mean (SD) or (SE))**									
	**Men**					**Women**				
	**No vertebral fracture (n=771)**	**Vertebral fracture, moderate (n=66)**	**Vertebral fracture, severe (n=55)**	***P *** **moderate**^**a**^	***P *** **severe**^**a**^	**No vertebral fracture (n=1079)**	**Vertebral fracture, moderate (n=80)**	**Vertebral fracture, severe (n=81)**	***P *** **moderate**^**a**^	***P *** **severe**^**a**^
FVC% predicted^b^	98.2 (15.3)	96.8 (17.7)	97.7 (17.6)	0.479	0.837	101.2 (15.8)	103.4 (18.3)	101.0 (17.9)	0.238	0.896
FEV_1_% predicted^b^	88.9 (16.9)	86.1 (20.5)	85.0 (22.5)	0.218	0.111	93.1 (17.4)	94.9 (20.7)	91.7 (21.0)	0.352	0.526
FEV_1_/FVC% predicted^a^	90.6 (10.8)	88.5 (11.7)	86.2 (14.5)	0.134	0.004	92.0 (9.1)	91.1 (10.5)	90.1 (10.4)	0.441	0.078
Adjusted^c^ values	(n=665)	(n=61)	(n=44)			(n=800)	(n=56)	(n=48)		
FVC (liter (SE))	4.22 (0.024)	4.20 (0.080)	4.26 (0.095)	0.827	0.714	2.99 (0.015)	3.10 (0.057)	2.90 (0.064)	0.056	0.209
FEV_1_ (liter (SE))	3.08 (0.021)	3.02 (0.069)	3.10 (0.083)	0.441	0.847	2.21 (0.013)	2.31 (0.050)	2.16 (0.056)	0.054	0.373
FEV_1_/FVC (SE)	0.73 (0.003)	0.72 (0.010)	0.73 (0.012)	0.215	0.821	0.74 (0.002)	0.75 (0.009)	0.74 (0.010)	0.402	0.973

The Tromsø Study 2007-08.

^a^Versus no fracture.

^b^Equation from Langhammer et al. [27].

^c^Adjusted for age, smoking habits, height, weight, physical inactivity, cardiovascular disease, lung disease, corticosteroids, hip BMD, hormones for menopause (women).

### Multiple adjusted FVC, FEV_1_, and FEV_1_/FVC values

When FVC, FEV_1_, and FEV_1_/FVC values were adjusted for multiple covariates, we found no significant associations with prevalent vertebral fracture, number of fractures, fracture site or severity.

### Obstructive and restrictive ventilatory impairment

When pulmonary dysfunction was characterized according to restrictive and obstructive ventilatory impairment, age-adjusted analyses showed that women and men with prevalent vertebral fracture had higher risk of severe obstructive ventilatory impairment than those with no vertebral fracture, odds ratio (OR) 5.18 in women (P = 0.029) and 2.72 in men (P = 0.003) (Table 6). However, multiple adjustments resulted in non-significant ORs. Prevalent vertebral fracture was not associated with mild or moderate obstructive or restrictive ventilatory impairment (results not shown).

**Table 6:** Odds for severe obstructive ventilatory impairment in relation to vertebral fracture

	**Severe obstructive pulmonary disease (FEV**_**1**_**% predicted <50% and FEV**_**1**_**/FVC <70%)**					
**Vertebral fracture**	**Men**			**Women**		
	**n (%)**	**Model 1**^**a **^**OR (95% CI)**	**Model 2**^**b **^**OR (95% CI)**	**n (%)**	**Model 1**^**a **^**OR (95% CI)**	**Model 2**^**b **^**OR (95% CI)**
No vertebral fracture	17 (2.2%)			8 (0.7%)		
Vertebral fracture	7 (5.8%)	2.72 (1.11-6.71)	1.41 (0.36-5.50)	6 (3.7%)	5.18 (1.77-15.14)	2.65 (0.53-13.14)

The Tromsø Study 2007-08.

^a^Model 1: Unadjusted.

^b^Model 2: Adjusted for smoking habits, weight, physical inactivity, cardiovascular disease, lung disease, corticosteroids, hip BMD.

In a model including a subsample of 859 men and 1199 women, current height was replaced by previous height. Adjusting for previous height did not significantly alter the results of the main model with current height.

## Discussion

In this cross-sectional study, we found few differences in predicted pulmonary values when comparing people with and without prevalent vertebral fracture. We noted, however, that in men, FVC% predicted, FEV1% predicted, and FEV_1_/FVC% predicted ratio decreased as the number of vertebral fractures increased. Furthermore, women and men with 4 or more fractures had significantly lower FVC% predicted than those without fracture. Multiple adjusted FVC, FEV_1_, and FEV_1_/FVC values were not significantly associated with vertebral fracture. Finally, presence of a vertebral fracture was not associated with mild, moderate, or severe obstructive or restrictive ventilatory impairment.

Previous studies have shown an association between vertebral fracture and reduced FVC% predicted and FEV_1_% predicted [14,16,17], which we did not replicate in our study. However, in contrast to our study, in which calculation of predicted values is based on current height, two of the previous studies based the calculations of predicted values on arm span [14] or recalled height [17]. When Schlaich et al. [17] used current height instead of recalled height, the associations became non-significant, consistent with our results.

Vertebral fracture may cause both height loss and impaired pulmonary function, thus adjustment for current height may bias the association toward null. Larger than normal height reduction, frequently presented in subjects with vertebral fracture, may overestimate lung function (in % of predicted) with adjustments for current height instead of previous height or arm-span [28]. We therefore performed analyses based on previous height measures from 14 years earlier in a subsample of the population, but we still found no associations between pulmonary function and vertebral fracture, as opposed to the associations found by Leech et al. [14] and Schlaich et al. [17].

In accordance with Leech et al. [14], who estimated that for each additional vertebral fracture, FVC% predicted was reduced by 9%, we found that FVC% predicted, as well as FEV_1_% predicted and FEV_1_/FVC% predicted ratio, decreased as number of vertebral fractures increased, although in men only. Unfortunately, kyphosis was not measured in our study.

Recently, Horie et al. [29] reported reduced FVC, FEV_1_ and FVC% predicted with increased lumbar lordosis angle, but not thoracic kyphosis angle, in elderly women. In our study, we found significantly lower FEV_1_/FVC% predicted in women and men with lumbar vertebral fracture than in those without fracture. This may seem surprising, as the lumbar vertebrae are not part of the rib cage. However, according to Horie et al. [29] there may be a reasonable theory behind these findings. The lumbar vertebrae are attached to the diaphragm, which main function is to contract and expand the lungs during inspiration. Lumbar vertebral fracture may lead to reduced constriction efficiency of the diaphragm and thus decrease the expansion of the lungs [29]. It should be noted that analyses of severity and number of fractures in men had low power due a small number of fractures.

There could be several explanations for the disparity between our study and previous studies, which are small patient studies, primarily including women [11,14,16,17]. Furthermore, some of the previous studies did not adjust for smoking status [14,17], which could be a confounding factor, due to the strong effect on lung function. Previous studies have identified vertebral fractures radiographically [14,16,17], whereas VFA with semi-quantitative analyses was used in our study. Recent developments in DXA technology now allow population-based identification of prevalent vertebral fracture using DXA densitometers [30]. Although spine radiographs are generally considered to be the gold standard for the diagnosis of vertebral fractures [31,32], the morphometric method used in our study is recognized to be easy and precise and with low radiation exposure [24,33,34]. The vertebral fracture assessment method has been used in many population settings, and its sensitivity and specificity are comparable to spinal radiographs in the ability to diagnose grade 2 and 3 vertebral fracture [8,30]. Still, different methods may produce disparities.

The multiple regression covariates are chosen based on previous research and possible confounding effects. As expected, age was the most important covariate, and the other covariates provided only minor changes to the results. There are few reports of the association between physical activity and vertebral fracture, but most studies show a reduced risk of vertebral fracture with higher physical activity levels [35-39]. Physical activity is also related to lung function, as COPD has been shown to limit physical capacity [40], although it is generally believed that ventilatory capacity does not limit performance in healthy humans. In our study, physical activity was not related to pulmonary function, probably because most subjects were healthy. Adjusting for cardiovascular (CVD) and lung disease (CLD) is based on the assumption that for instance CVD could be a confounder in the relationship between lung function and vertebral fracture. When BMD is measured at the hip, it has been shown that relative risk of vertebral fractures increases 1.6 times per SD [41]. In our study, men and women have similar rates of fractures, although women have lower BMD in the hip. This may indicate that fact that fracture risk is also affected by many other factors, such as other skeletal mechanisms (bone architecture and geometry) [42].

Limitations of this study include the cross-sectional design, which prevents us from determining any cause-effect associations. We do not know how or when the identified vertebral fractures occurred. Many vertebral fractures are asymptomatic [43], others may be of mechanic origin or may have happened many years ago; all are factors that may weaken the association between prevalent vertebral fractures and pulmonary function. Moreover, we hypothesized that the presence of a vertebral fracture imposes restrictions on the pulmonary function, based on the assumption that the mechanisms underlying an association involve spinal kyphosis, which leads to decreased thoracic volume and disturbances in the joint between the vertebral body and the rib and the respiratory muscles [17,29]. Reversing the cause-effect direction, some previous case–control studies have shown that patients with COPD may be at higher risk for osteoporosis and vertebral fractures [44], through mechanisms such as inflammation [45], increased bone resorption [46] or steroid use [47]. Osteoporosis may in turn increase the risk of vertebral fracture and further exacerbate pulmonary dysfunction, creating a negative circle.

Our observational design opens for selection bias. Although the Tromsø Study has a high participation rate, individuals with poor health may be under-represented. In the context of a previous Tromsø Study [48], a survey among non-participants showed that the responders were less likely to be smokers and to have respiratory symptoms, but more likely to be married, than the non-responders, whereas age, body mass index, and blood pressure did not differ. Although differences between participants and non-participants should be a matter of concern to the validity, we believe the association between vertebral fracture and pulmonary function most likely did not differ between the participants in our study and eligible persons who did not attend. For further assessment of the impact of vertebral fracture on pulmonary function, longitudinal studies are warranted.

## Conclusion

Although we found an inverse association between some pulmonary markers and number of vertebral fractures, we conclude that this study identified limited indications of any clinically relevant associations between vertebral fracture and ventilatory impairment in elderly men and women.

## Abbreviations

ANCOVA: Analysis of covariance; ANOVA: Analysis of variance; BMD: Bone mineral density; COPD: Chronic obstructive pulmonary disease; DXA: Dual X-ray; FEV1: Forced expiratory volume in one second; FVC: Forced vital capacity; OR: Odds ratio; VFA: Vertebral fracture assessment.

## Competing interests

The authors declare that they have no competing interests.

## Authors’ contributions

NE made substantial contributions to the conception of the study. BM, SW and HM participated in the design and interpretation of data; BM, MRT, HM and MJR participated in the analysis and interpretation of data. SW did the DEXA scans and carried out the vertebral fracture analyses. BM performed the statistical analyses and drafted the manuscript. All authors helped revising the manuscript and all approved the final version to be published.

## Pre-publication history

The pre-publication history for this paper can be accessed here:

http://www.biomedcentral.com/1471-2318/13/116/prepub
